# Reducing patients’ suicide ideation through training mental health teams in the application of the Dutch multidisciplinary practice guideline on assessment and treatment of suicidal behavior: study protocol of a randomized controlled trial

**DOI:** 10.1186/1745-6215-14-372

**Published:** 2013-11-06

**Authors:** Derek P de Beurs, Marieke H de Groot, Judith E Bosmans, Jos de Keijser, Jan Mokkenstorm, Bas Verwey, Erik van Duijn, Remco FP de Winter, Ad JFM Kerkhof

**Affiliations:** 1 Department of Clinical Psychology, VU University, Amsterdam, The Netherlands; 2 EMGO Institute for Health and Care Research, Amsterdam, The Netherlands; 3 Department of Health Sciences, Faculty of Earth and Life Sciences, VU University, Amsterdam, The Netherlands; 4 GGZ Foundation for Mental Health Care, Friesland and Groningen University, Groningen, The Netherlands; 5 GGZ Foundation for Mental Health Care, GGZ inGeest, 113Online Foundation, Amsterdam, The Netherlands; 6 Rijnstate Hospital, Arnhem, The Netherlands; 7 GGZ Foundation for Mental Health Care, Delfland, The Netherlands; 8 GGZ Foundation for Mental Health Care, Parnassia, The Hague, The Netherlands

**Keywords:** Suicide prevention, Suicidal behavior, Guideline application, Implementation, Cost-effectiveness, Cost-utility, Multicentre, E-learning, Train-the-trainer, Professionals

## Abstract

**Background:**

To strengthen suicide prevention skills in mental health care in The Netherlands, multidisciplinary teams throughout the country are trained in the application of the new Dutch guideline on the assessment and treatment of suicidal behavior. Previous studies have shown beneficial effects of additional efforts for guideline implementation on professionals’ attitude, knowledge, and skills. However, the effects on patients are equally important, but are rarely measured. The main objective of this study is to examine whether patients of multidisciplinary teams who are trained in guideline application show greater recovery from suicide ideation than patients of untrained teams.

**Methods/Design:**

This is a multicentre cluster randomized controlled trial (RCT), in which multidisciplinary teams from mental health care institutions are matched in pairs, and randomly allocated to either the experimental or control condition. In the experimental condition, next to the usual dissemination of the guideline (internet, newsletter, books, publications, and congresses), teams will be trained in the application of the guideline via a 1-day small interactive group training program supported by e-learning modules. In the control condition, no additional actions next to usual dissemination of the guideline will be undertaken.

Assessments at patient level will start when the experimental teams are trained. Assessments will take place upon admission and after 3 months, or earlier if the patient is discharged. The primary outcome is suicide ideation. Secondary outcomes are non-fatal suicide attempts, level of treatment satisfaction, and societal costs. Both a cost-effectiveness and cost-utility analysis will be performed. The effects of the intervention will be examined in multilevel models.

**Discussion:**

The strengths of this study are the size of the study, RCT design, training of complete multidisciplinary teams, and the willingness of both management and staff to participate.

**Trial registration:**

Netherlands trial register: NTR3092

## Background

To strengthen suicide prevention in mental health care in The Netherlands, the Dutch multidisciplinary practice guideline on the assessment and treatment of suicidal behavior (PGSB) has been developed by representatives from the Dutch Association of Psychiatrists (NVvP), the Dutch Association of Psychologists (NIP), the Dutch Nurses’ Association (V&VN), and supported by the Dutch Knowledge Centre on Mental Health and Addiction (Trimbos Institute) [1]. The PGSB combines the stress-diathesis model [2] and the entrapment model [3] to explain the onset of suicidal behavior. It consists of chapters on the theoretical concept of suicidal behavior, basic assumptions of professional practice (fostering a therapeutic alliance with the suicidal patient, providing continuity of care and safety, and a systematic assessment of suicidal behavior), treatment of suicidal behavior, and professional practice following a suicide. Importantly, suicidal behavior is considered the focus of treatment. The guideline was issued in May 2012 [1].

One consistent finding in the literature is the gap between guideline development and the application of guidelines in daily health care practice [4-6]. As a consequence, patients do not always receive appropriate care [6,7]. Grol and Grimshaw (2003) suggested that structured implementation can improve adherence to guidelines [6]. Despite its importance, implementation science is still just emerging [8]. Theory-based and tailored implementation approaches are widely developed and studied [9] but no ‘magic bullet’ [10] to improve health care has been found to date. Knowledge of effective strategies is limited, whether from highly controlled studies with limited external validity, or from field studies with no significant effect or small effect sizes. Moreover, patient outcomes are rarely assessed in implementation studies. Regarding implementation in psychiatry, two systematic reviews [11,12] showed a modest effect of implementation of psychiatric guidelines on care and patient outcome, and concluded that there is a need for more studies on the effects of guideline implementation at both a patient and professional level. Overall, there is limited empirical evidence on the most effective strategy to implement guidelines in general [6,8,9], in particular in mental health care [11,12].

Considering the importance of the PGSB, the evidence on non-adherence to guidelines, and the lack of evidence-based implementation strategies, the Dutch government commissioned a study on the effectiveness of the implementation of the PGSB. In 2011, the PITSTOP suicide (professionals in training to stop suicide) study started to examine the effect of a multifaceted, e-learning supported train-the-trainer implementation (TtT-e) program delivered to multidisciplinary teams of mental health care departments in a randomized cluster trial.

The content of the TtT-e program is based on the PGSB. In the TtT-e, senior staff members are trained by suicide experts. Subsequently, trained staff members train their multidisciplinary teams, using role play and personalized feedback.

The effectiveness of the TtT-e program on guideline implementation is expected since research has shown that small group training on suicidal behavior assessment with role play and personalized feedback leads to improved professional confidence and effective professional behavior [13]. Also, since suicide prevention in mental health care is essentially multidisciplinary, professionals are trained in multidisciplinary groups [4]. The training is supported by an e-learning module as e-learning is considered to complement face-to-face training in medical settings; Ruiz *et al*. reported that e-learning helped medical students become more actively involved in the study material, and thereby helped to internalize the material [14].

The PITSTOP suicide study aims to evaluate the effect of the TtT-e program at both a patient and professional level. The design of the study at the professional level has already been published [15] and the outcomes will be presented in a separate article. This article describes the protocol for the patient-level study.

The TtT-e program aims to strengthen the competences of health care professionals. The primary outcome of the present study is suicide ideation of patients due to improvement of professional skills. Professional and gatekeeper training in diagnosis and treatment of depressive disorders, which are associated with suicidal behavior [16], have been shown to result in a reduction of suicide rates when delivered to general practitioners [17-19]. A reduction of self-destructive acts in adolescents of an American Indian Tribal Nation was found after a suicidal behavior program [20], and a suicide prevention program in the US Air Force personnel resulted in a decline of the suicide rate [21]. However, the effects of professional or gatekeeper training programs on suicide rate and suicidal behavior are investigated in non-randomized controlled study designs. As a result, the research conducted to date does not clearly demonstrate whether professional or gatekeeper training has unique and independent effects on actually reducing suicidal behavior.

Due to increasing budget constraints and rising costs of mental health care, evidence on effectiveness alone is not sufficient for policy making. Policy makers aim to maximize health benefits from the budget available and, therefore, need information on both the costs and effects of the interventions. Thus, an economic evaluation to provide this information will also be performed. Most economic evaluations of guideline implementation strategies have methodological deficits and do not consider all relevant costs and benefits [22]. In the current study, we will estimate both the treatment and policy cost-effectiveness of the TtT-e program in comparison with usual care, as described by Mason *et al*. [23]. We will report the outcomes of the economic evaluation in a separate article.

In sum, the primary outcome of the current study is recovery from suicidal ideation. We hypothesize that patients treated by multidisciplinary teams who are trained by the TtT-e program will recover more quickly from suicidal ideation as compared with patients treated by multidisciplinary teams who were not trained.

Secondary outcomes are non-fatal suicide attempts and satisfaction with treatment. We hypothesize that patients in the intervention condition will report fewer non-fatal suicide attempts and more satisfaction with treatment than patients in the control condition.

A further aim of this study is to evaluate the cost-effectiveness and cost-utility of the intervention. We hypothesize that the intervention will be more cost-effective compared with implementation as usual (IAU).

## Method

### Design

This is a multicentre cluster randomized controlled trial (RCT) in which multidisciplinary teams from mental health institutions (MHIs) are matched in pairs with respect to patient diagnoses and average treatment duration. Subsequently, pair members are randomly allocated to treatment conditions.

A MHI is a regional organization with a specific catchment area of patients [24]. For example, in 2011, MHI Rivierduinen had a catchment area of 1.1 million people within an area of approximately 1,500 km^2^ in the region of South Holland. MHI Rivierduinen has 44 psychiatric departments and treats an overall number of 24,753 patients annually with 2,726 employees [25]. Most of the psychiatric departments are specialized in the treatment of patients of specific diagnostic categories, such as depression or personality disorder.

### Recruitment

**Figure 1:**
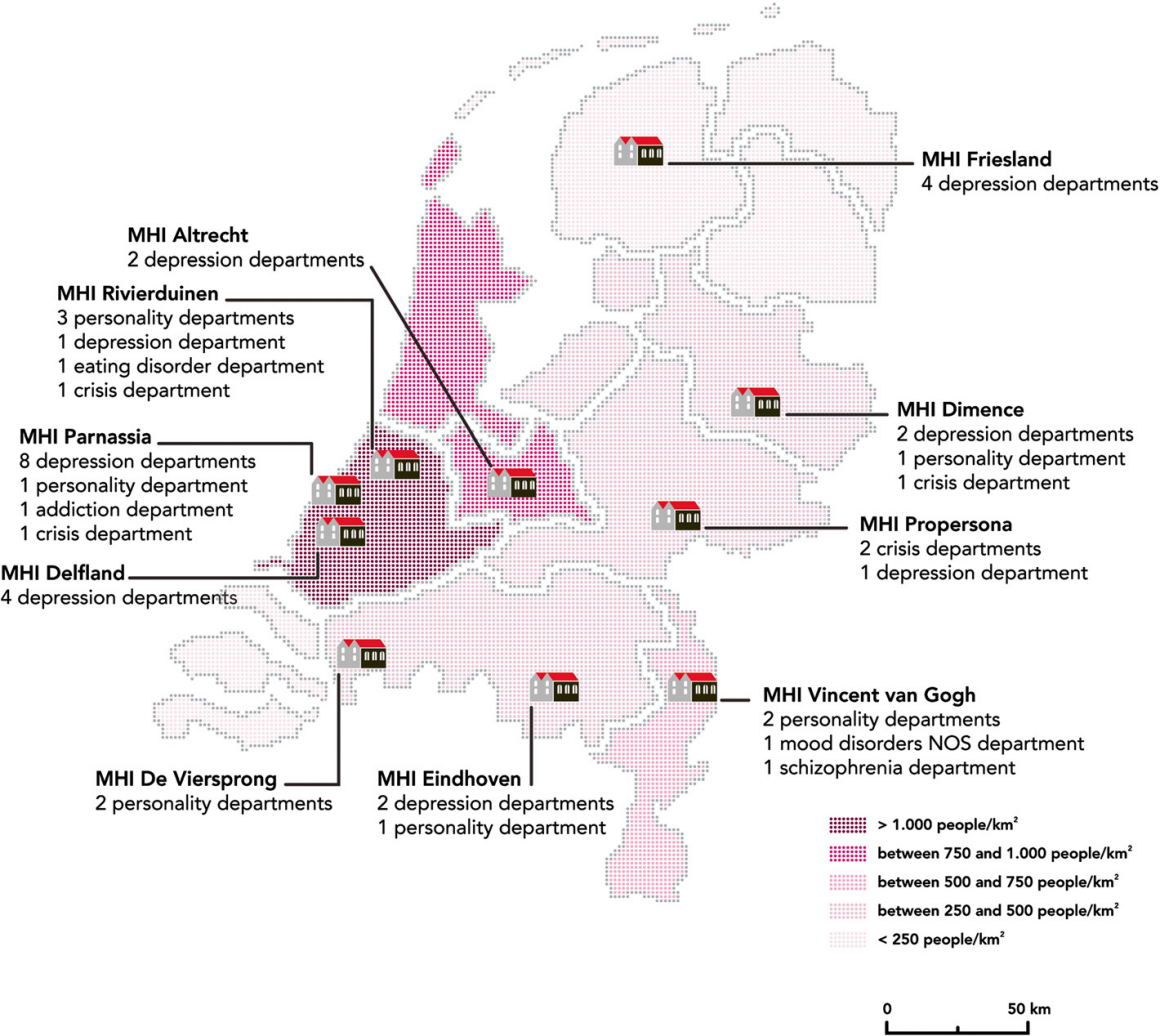
**Overview of the ten MHIs.** The number of departments and the primary patient group per department are indicated. MHI, mental health institution.

Recruitment of MHIs took place during meetings and conferences on suicide prevention in The Netherlands between 2008 and 2010. MHIs were invited to participate in the study. When MHIs expressed willingness to participate, they were requested to indicate at least two multidisciplinary departments for adult care to be included in the study. To prevent exchange of training materials between the experimental and control condition, MHIs were explicitly asked to provide departments that were based in separate buildings and/or on separate locations, and to be sure that staff members were not exchanged or shared between departments. This resulted in 43 participating departments distributed over 10 MHIs (Figure 1). Various types of mental health care departments are represented in the study (inpatient and outpatient care, crisis departments, and long-stay departments) treating patients of various diagnostic categories (personality disorder, depressive disorder, anxiety disorder, and psychotic disorder) and of various ages.

### Measurement procedure

**Figure 2:**
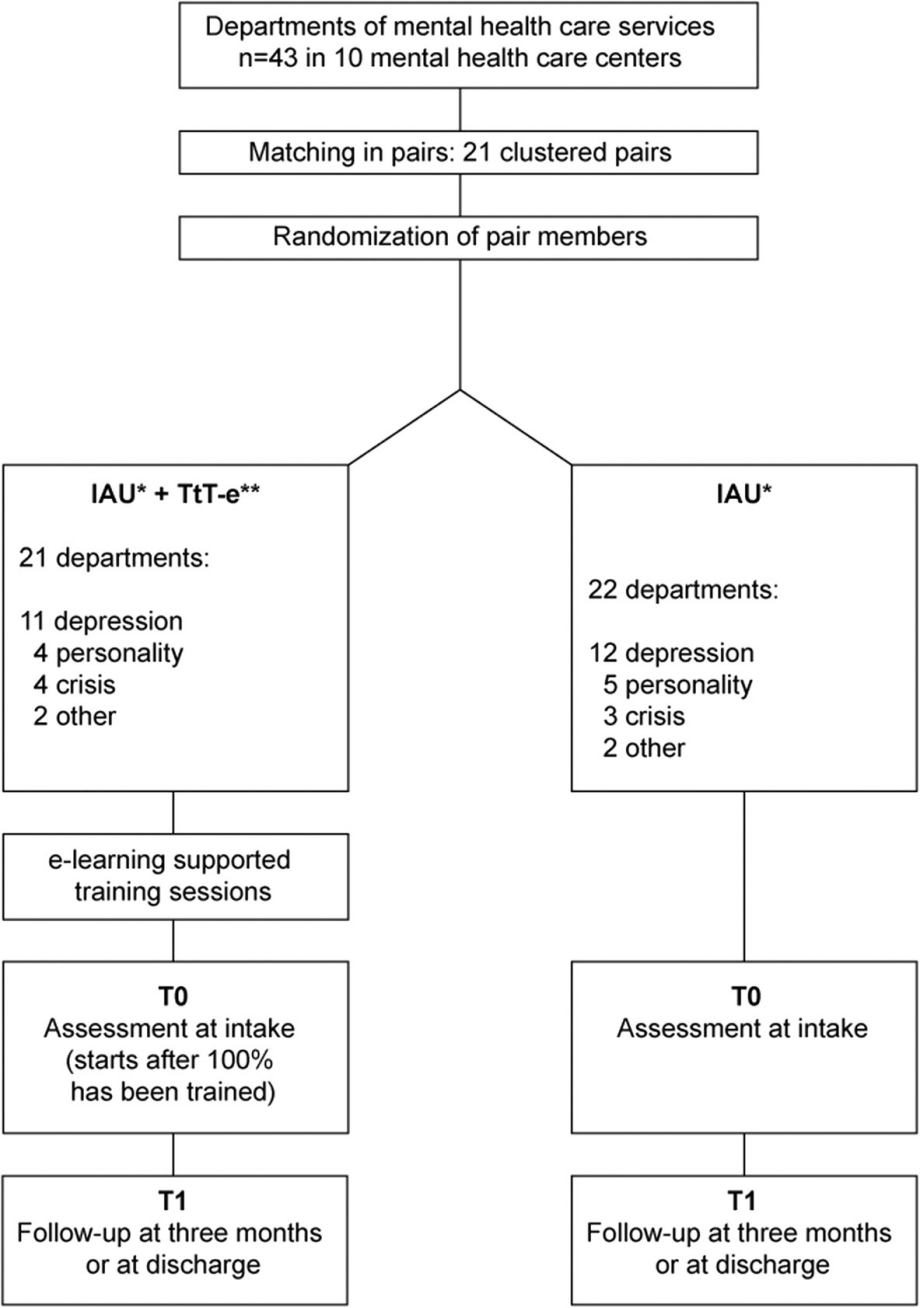
**PITSTOP suicide study design.** IAU, implementation as usual; PITSTOP suicide, professionals in training to STOP suicide; T0, admission; T1; 3 months after admission; TtT-e, e-learning supported train-the-trainer implementation.

At patient level, the preferred mode of data collection is by routine outcome monitoring (ROM), an online system by which data on the effectiveness of treatment in everyday clinical practice are systematically collected [26]. In MHIs not using ROM, graduate students and/or research assistants will use paper and pencil questionnaires to collect data. The results from the graduate students/research assistants are expected to be comparable to the data collected via ROM. ROM also works with assistants to collect data and help participants to understand items/instructions [26]. Using differential item functioning (DIF) [27], we will investigate if the findings are indeed comparable, and control for any bias due to difference in data collection in the final analysis. By collecting data either via the ROM or graduate students/research assistants, we aim to reach all newly admitted patients in the participating departments. As the intervention focuses on fostering a working relationship with suicidal patients, the intervention is expected to be most effective during the first month of treatment. Therefore, patients will be assessed directly at admission (T0) and at 3 months after admission (T1) (Figure 2). If a patient is discharged within 3 months, T1 will be arranged just before discharge. Measurements in the experimental departments will start immediately after all staff are trained. In the control departments, T0 will start when the department is informed of the allocation outcome. All eligible patients will be informed about the study and will be asked to provide written informed consent.

### Measurement at patient level

#### Suicide ideation and suicide attempts

The primary outcome, suicide ideation, will be measured using the Beck Scale for Suicidal Ideation (BSS) [28], a well-validated and widely used instrument. The BSS consists of 21 self-reported items. The first 19 items measure the severity of actual suicidal wishes and plans; item 20 assesses the number of previous suicide attempts; and item 21 assesses the severity of the last suicide attempt. Normally, if a patient scores 0 on items 1 through 5, items 6 through 19 are automatically scored 0, and the patient is directed to item 20. If the patient scores more than 0 on items 1 through 5, all items of the BSS are completed. In our study we asked patients to complete all 19 items, even if they scored 0 on items 1 through 5. By doing so, we hoped to be able to detect small differences in suicide ideation in patients with a low suicide trait. The overall score is computed by summing the scores of the first 19 items. The overall score ranges from 0 to 38; a higher score indicates a higher level of suicide ideation. Item 20 and 21 are not used to calculate the ideation score, but are used to assess the number and intensity of previous suicide attempts.

#### Treatment satisfaction

Treatment satisfaction will be assessed with four items established to measure the quality of therapeutic alliance. The first two items are: ‘How satisfied are you with your therapy?’ and ‘How would you evaluate your relationship with your therapist?’. These two items are rated from 0 to 10. Next, we will assess treatment satisfaction with reference to patients’ suicidal behavior: ‘Was there any attention for your suicidal thoughts during therapy?’ and ‘How did your therapist deal with your suicidal thoughts?’. These two items are scored on a four-point Likert scale ranging from: 1, very well, to 4, very poor. As the questions focus on the patient’s experience, these four items will only be administered at T1.

#### Cost-effectiveness

Costs incurred by patients during the course of the study will be measured with an adapted version of the Trimbos questionnaire for costs associated with psychiatric illness (TiC-P) [29]. The TiC-P consists of two parts: part one measures direct medical costs (for example visits to a psychiatrist or a psychologist) and part two measures indirect costs (for example costs due to sick leave and productivity losses while being at work but not functioning optimally). If available, Dutch guideline prices will be used to value resource use [30]. Medication use will be valued using prices of the Royal Dutch Pharmaceutical Society. Lost productivity costs will be calculated according to the friction cost approach (friction period 154 days) using the mean age- and sex-specific income of the Dutch population [30]. Costs of the development of the intervention will be based on the salaries of the researchers for the development of the TtT-e program, material costs, implementation costs of the e-learning environment, and the costs of the production losses due to training of multidisciplinary teams.

#### Quality of life

Quality of life will be measured using the EQ-5D (EuroQol, Rotterdam, The Netherlands) [31]. This is a five-item questionnaire with an additional visual analogue scale (VAS) developed to assess health-related quality of life. The five items represent the dimensions of mobility, self-care, usual activities, pain/discomfort, and anxiety/depression. Items are scored on a three-point Likert scale: 1, no problem; 2, some problems; and 3, extreme problems. The health states obtained from EQ-5D will be converted to utility scores using the Dutch EQ-5D tariff. Quality-adjusted life years (QALYs) will be calculated using the area-under-the-curve method with linear interpolation between time points. A VAS is also included to measure the patient’s self-rated health from: 1, best imaginable health state, to 5, worst imaginable health state.

### Inclusion and exclusion criteria

Departments are considered eligible for participation if they treat patients aged ≥18 years and if professionals consider a need for training in suicide prevention skills, and their management are willing to provide support including financial support for covering loss of production.

Since basic clinical skills, such as establishing a therapeutic alliance with the patient, are an important part of the training and e-learning, it is expected that patients who report no suicide ideation at baseline will benefit from the TtT-e program. Therefore, all patients irrespective of being suicidal at baseline will be included. Since admitted patients will often be affected by emotional and/or cognitive problems, patients who are emotionally and/or cognitively unable to complete questionnaires properly will be excluded. Whether a patient is able to enter the study will be left to the discretion of the staff.

### Randomization procedure

Eligible departments were clustered in pairs. It is assumed that similar types of patients treated for a similar duration of time are likely to be comparable. The first criterion for matching departments was the main diagnostic category of patients. For instance, a department that reported that 60% of the patients had a main diagnosis of depression was matched with another department that reported a comparable percentage of depressive patients. Within groups of comparable patient diagnoses, departments were matched with departments with comparable average treatment duration of patients. Members of matched pairs were randomly allocated to either IAU (internet, newsletter, books, publications, and congresses; control) or IAU with the TtT-e program (intervention). Randomization was performed by an independent researcher of the research institution (EMGO + Institute for Health and Care Research, Amsterdam, The Netherlands) who was not involved in the study. Table 1 displays an overview of the types of departments, patient diagnoses, and results of the matching procedure. Healthcare professionals are aware of the allocation outcome, but patients are not.

**Table 1:** Overview of the mental health institutions (MIHs), departments, main patient diagnoses, and results of the matching procedure

**MHI**	**Department**	**Average treatment duration of patients (days)**	**Main diagnosis**	**Condition**
Delfland	Inpatient	1,532	Depression	EXP
Delfland	Inpatient	1,308	Depression	CON
Delfland	Outpatient	624	Depression	EXP
Dimence	Outpatient	600	Depression	CON
Parnassia	Outpatient	21	Depression	CON
Parnassia	Inpatient	16	Depression	EXP
Delfland	Outpatient	740	Depression	CON
Parnassia	Inpatient	730	Depression	EXP
Pro Persona	Crisis	1	Depr/bord/crisis	CON
Pro Persona	Crisis	1	Depr/bord/crisis	EXP
Parnassia	Inpatient	250	Personality	EXP
Dimence	Inpatient	400	Personality	CON
Dimence	Crisis	1	Depr/bord/crisis	EXP
Parnassia	Crisis	1	Depr/bord/crisis	CON
Friesland	Outpatient	582	Depression	EXP
Friesland	Outpatient	814	Depression	CON
Friesland	Outpatient	743	Depression	EXP
Friesland	Outpatient	602	Depression	CON
Parnassia	Outpatient	90	Depression	EXP
Parnassia	Outpatient	21	Depression	CON
Viersprong	Personality	240	Personality	CON
Eindhoven	Personality	300	Personality	EXP
Eindhoven	Outpatient	150	Depression	CON
Eindhoven	Inpatient	104	Depression	EXP
Rivierduinen	Inpatient	35	Depression	EXP
Rivierduinen	Outpatient	240	Depression	CON
Parnassia	Outpatient	41	Depression (older adults)	EXP
Parnassia	Outpatient	Missing	Depression (older adults)	CON
Dimence	Inpatient	90	Depression	CON
Pro Persona	Outpatient	365	Depression	EXP
Altrecht	Outpatient	42	Depr/bord/crisis	EXP
Altrecht	Inpatient	150	Depr/bord/crisis	CON
Vincent van Gogh	Outpatient	172	Personality	CON
Vincent van Gogh	Outpatient	54	Personality	EXP
Rivierduinen	Inpatient	270	Personality	CON
Rivierduinen	Outpatient	180	Eating disorder	EXP
Viersprong	Outpatient	244	Personality	EXP
Parnassia	Outpatient	240	Addiction	CON
Rivierduinen	Crisis	Missing	Depr/bord/crisis	EXP
Rivierduinen	Inpatient	Missing	Personality	CON
Vincent van Gogh	Inpatient	96	Mood disorder NOS	CON
Vincent van Gogh	Inpatient	20	Schizophrenia	EXP
Parnassia	Inpatient	371	Depression (older adults)	CON

CON, control; Depr/bord/crisis, Depression/borderline/crisis; EXP, experimental; personality, personality disorder; MIH, mental health institution; missing, missing data; NOS, not otherwise specified.

### Intervention

In the experimental condition, the complete multidisciplinary teams (all registered nurses, psychologists, physicians, and psychiatrists) will be trained in the application of the guideline via the TtT-e program. By training complete multidisciplinary teams, including all team members, irrespective of full- or part-time staff, we aim for 100% coverage. In the TtT-e program, three types of actors are involved: masters, trainers, and trainees. The training is applied on two levels: first, trainers are trained by masters. Subsequently trainees are trained by trainers. The training program is supported by two e-learning modules. The first module is developed for trainees. It consists of video vignettes in which experienced nurses, psychologists, and psychiatrists interact with suicidal patients (played by actors). The total running time of this module for trainees is 60 minutes. In addition to the e-learning module for trainees, a second e-learning module was developed specifically for trainers. It provides a video of the first training session provided by masters to trainers, which was processed into an e-learning format allowing trainers to review the training session.

The TtT-e is a 1-day small interactive group program supported by e-learning modules, which reflects the PGSB recommendations [1]. The PGSB recommendations served as the starting point to set the content of the TtT-e program within the PITSTOP suicide study. First, all guideline recommendations were listed and clustered into six themes: 1) basic clinical skills when discussing suicidality; 2) systematic assessment of suicidal behavior; 3) diagnosis of the current suicidal condition; 4) safety and continuity of care, including participation of the patient’s relatives; 5) treatment of suicidal behavior; and 6) chronic suicidal conditions. Second, clusters were transformed into six modules and scheduled according to the sequence of action in common clinical practice. For each module, goals and competences were set in terms of professional behavior. Furthermore, the PGSB recommends systematic investigation of the suicidal condition of patients by using the Chronological Assessment of Suicidal Events (CASE) interview [32]. Based on its outcome, risk and protection factors for suicide of individual patients are weighted. Subsequently, structured diagnosis, treatment strategy, and a safety protocol are determined. In the TtT-e program, the CASE interview is the overall framework for each of four role plays in which one trainee acts as a suicidal patient and the other trainee interviews the ‘patient’ via the CASE interview.

In the control condition, no additional actions next to IAU will be undertaken.

To survey adherence to the training program by trainers, graduate students will randomly visit training sessions, and will rate adherence on a four-point Likert scale: 1, very strong adherence, to 4, very low adherence.

### Sample size

For the primary outcome (suicide ideation) we calculated the effect size according to the recommendations of Twisk [33]. The number of patients that needed to be included was set to 423. This number is sufficient to find a small effect size (Cohen’s *d*) of 0.3, assuming an alpha of 0.05 and statistical power of 1-beta = 0.80. A correction of 20% for clustering of effects within departments is applied. An average number of 20 patients per department will be included.

#### Statistical analyses

As the study is a design with different levels, all outcomes will be analyzed using multilevel models. Patients are nested within departments (level 1), and departments are nested within MHI centers (level 2). Multilevel models are hierarchal systems that estimate random coefficients and variance components for each level [33]. Random intercepts will be included in the multilevel models. Data will be analyzed on an intention-to-treat basis.

To examine the hypothesis that suicide ideation in patients of professionals who received the TtT-e program is more likely to be reduced than patients of professionals who did not receive TtT-e, we will compare changes in BSS scores between patients of teams allocated to the intervention with patients of teams in the control group. To examine differences in the number and intensity of suicide attempts, scores on items 20 and 21 of the BSS will be dichotomized (YES/NO, LOW/HIGH) and compared between treatment groups using logistic regression. To examine the effect of the intervention on treatment satisfaction, mean scores of the four satisfaction items between groups will be compared. For the economic evaluation, missing cost and effect data will be imputed using multiple imputation according to the MICE algorithm [34]. The results of the imputed datasets will be pooled using Rubin’s rules [35]. Bias-corrected and accelerated bootstrapping with 5,000 replications will be used to estimate 95% confidence intervals around the mean difference in total costs between the treatment groups. Incremental cost-effectiveness ratios (ICERs) will be calculated by dividing the difference in mean total costs between the treatment groups by the difference in mean effects between the treatment groups. Bootstrapping will also be used to estimate the uncertainty surrounding the ICERs, which will be graphically presented on cost-effectiveness planes. Cost-effectiveness acceptability curves will also be estimated. Cost-effectiveness acceptability curves will show the probability that the intervention is cost-effective in comparison with usual care for a range of different ceiling ratios (that is, the willingness to pay for one extra unit of effect) [36].

Approval was obtained from the Medical Ethics Committee of the VU University Medical Centre, Amsterdam, The Netherlands (registration number: 2011/151). All local medical ethics committees agreed with this approval.

## Discussion

This article describes the study protocol of a multicentre cluster RCT examining the additional effect of a multifaceted TtT-e program on suicide ideation with usual guideline implementation (IAU). Secondary outcomes are number and intensity of non-fatal suicide attempts, treatment satisfaction, cost-effectiveness, and cost-utility.

We hypothesize that, as a result of the improved skills and confidence of health care professionals due to IAU plus the TtT-e program, suicidal patients will recover more quickly from suicidal ideation and will show fewer suicide attempts as compared with patients in the control condition. Also, we hypothesize that patients from departments allocated to the experimental condition will show more treatment satisfaction. Additionally, we expect that IAU plus the TtT-e program will be more cost-effective compared to IAU.

To date, the evidence on effective strategies to implement guidelines in mental health care is scarce. If the study hypotheses are confirmed, the TtT-e program could be more widely distributed and applied in mental health care, reducing suicidal behaviors and limiting the costs due to suicidal behaviors.

A strength of this study is that data are collected in a naturalistic setting and hypotheses are examined in a RCT design. A further strength is the size of the study in which various MHIs and patients of miscellaneous diagnostic categories are included. We suggest that the patient flow within these 43 departments fairly represents the Dutch patients in mental health care. Of the 30 large MHIs in The Netherlands that were approached for the study, 10 were included in the study. From the many hundreds of psychiatric departments within these 10 MHIs, 43 departments joined the study. Patient diagnoses range from affective disorders, personality disorder, to addiction disorder. Among the 43 departments are long-stay, crisis, and inpatient and outpatient departments. The age of patients range from 18 to over 80 years. Figure 1 demonstrates that the included departments are located in urban and rural regions of The Netherlands. We conclude that the included departments provide a representative sample of all departments in mental health care institutions in The Netherlands. We will compare the patient characteristics of the sample with the national patient population available via the Dutch Association of Mental Health and Addiction Care (GGZ Nederland, Amersfoort, The Netherlands), and will describe the results to check external validity.

A possible limitation of the study is that budget cuts, reorganizations, and layoffs, commonplace in Dutch mental health care, may interfere with the study aims. Attrition of patients to the study is a further challenge, since not all patients will complete the ROM at all intervals due to the lack of time of professionals or inexperience with the ROM [37]. Using a strict protocol and local research assistants/nurses, we aim for limited drop-out. To prevent drop-out, research assistants will help patients to complete the questionnaires and will make appointments for follow-up assessment.

Commitment of both management and employees of the participating MHIs is crucial. To date, willingness of both management and employees to apply the TtT-e program is excellent. The findings from the current study may be generalized to implementation of other (mental) health care guidelines.

## Trial status

Ongoing patient recruitment.

## Abbreviations

BSS: Beck Scale for Suicidal Ideation; CASE: Chronological Assessment of Suicidal Events; DIF: differential item functioning; IAU: implementation as usual; ICER: incremental cost-effectiveness ratio; MHI: Mental health institution; NIP: Dutch Association of Psychologists; NVvP: Dutch Association of Psychiatrists; PGSB: Practice guideline on the assessment and treatment of suicidal behavior; PITSTOP suicide: Professionals in training to STOP suicide; QALY: Quality-adjusted life year; RCT: Randomized controlled trial; ROM: Routine outcome monitoring; TiC-P: Trimbos questionnaire for costs associated with psychiatric illness; TtT-e: E-learning supported train-the-trainer implementation; V&VN: Dutch Nurses’ Association; VAS: Visual analogue scale.

## Competing interests

The authors declare that they have no competing interests.

## Authors’ contributions

AK, MdG, JM, and JdK obtained funding for this study. DdB, AK, MdG, JEB, and JdK, drafted the manuscript. DdB will undertake the study. MdG and AK designed the training protocol. EvD, RdW, BV, JEB, and JM participated in the design of the study, development of the intervention, and measurement procedure of patients. All authors read and approved the final manuscript.
